# The Conditions Necessary for the Formation of Dissipative Structures in Tribo-Films on Friction Surfaces That Decrease the Wear Rate

**DOI:** 10.3390/e25050771

**Published:** 2023-05-08

**Authors:** Iosif S. Gershman, German Fox-Rabinovich, Eugeniy Gershman, Alexander E. Mironov, Jose Luis Endrino, Pavel Podrabinnik

**Affiliations:** 1 Joint Stock Company Railway Research Institute, Moscow 3rd Mytischinskaya Street 10, 107996 Moscow, Russia; isgershman@gmail.com (I.S.G.);; 2 Department of Mechanical Engineering, McMaster Manufacturing Research Institute (MMRI), McMaster University, Hamilton, ON L8S 4L8, Canada; gfox@mcmaster.ca; 3 LLC «TransTriboLogic», Skolkovo Innovation Center, Bulvar Bolshoy 42 Build. 1, Office 337, 121205 Moscow, Russia; gershmanei@gmail.com; 4 Department of Engineering, Universidad Loyola Andalucia, Av de las Universidades s/n, 41704 Seville, Spain; 5 Laboratory of Electric Currents Assisted Sintering Technologies, Moscow State University of Technology “STANKIN”, Vadkovsky Lane 3a, 127055 Moscow, Russia

**Keywords:** friction, tribo-films, entropy production, self-organization, Ziegler’s principle, friction, wear, current collection, plain bearings, cutting

## Abstract

Tribo-films form on surfaces as a result of friction and wear. The wear rate is dependent on the frictional processes, which develop within these tribo-films. Physical–chemical processes with negative entropy production enhance reduction in the wear rate. Such processes intensively develop once self-organization with dissipative structure formation is initiated. This process leads to significant wear rate reduction. Self-organization can only occur after the system loses thermodynamic stability. This article investigates the behavior of entropy production that results in the loss of thermodynamic stability in order to establish the prevalence of friction modes required for self-organization. Tribo-films with dissipative structures form on the friction surface as a consequence of a self-organization process, resulting in an overall wear rate reduction. It has been demonstrated that a tribo-system begins to lose its thermodynamic stability once it reaches the point of maximum entropy production during the running-in stage.

## 1. Introduction

Protective layers of tribo-films form on surfaces during friction. It has been demonstrated that the overall wear rate is influenced by processes that develop within these layers [1,2,3]. Since energy transformation during friction occurs under far-from-equilibrium conditions, these processes should be analyzed on the basis of non-equilibrium thermodynamics [4,5,6,7]. It is shown in [8] that the wear rate of rubbing bodies changes in accordance with entropy production processes that take place within the tribo-films that form on the friction surface. This is consistent with [9].

A minimal wear rate can be reached when the entropy production is at a minimum. To predict friction parameters and to select the optimal friction modes, an observable regular pattern of entropy production change similar to that predicted by Prigogine’s theorem would be useful for processes occurring at far-from-equilibrium states. However, Prigogine’s theorem of the minimum production of entropy in a stationary state [10] is only valid in a region where the thermodynamic flows are strictly linearly dependent on thermodynamic forces (i.e., a linear region). Frictional processes, on the other hand, develop in strongly non-equilibrium regions [4,5,6,7,8,11,12,13,14,15,16,17]. They either do not obey linear laws or are not strictly linear, which means that Prigogine’s theorem does not apply to them. The absence of such mathematically established relationships for processes occurring during friction makes them poorly predictable, with the values of wear rate often having a significant deviation. This can be accomplished by applying the principles of minimum or maximum production of entropy in a non-equilibrium state, which are already widely known in the literature [18,19]. 

The discussions of these principles of non-equilibrium thermodynamics, in particular their application to living and non-living systems, were considered in [20,21,22,23,24,25,26,27,28,29,30,31,32,33,34,35,36,37,38,39,40,41,42,43,44]. However, the application of the principles of maximum and minimum entropy production to real, empirical processes remains a subject of open discussion [8]. A decrease in the production of entropy of processes, which occurs within the surface layers, can be achieved as a consequence of self-organization [45].

Self-organization, according to [28] constitutes the formation of dissipative structures. Dissipative structures are characterized by processes with negative entropy production [7,13,28,45]. At the same time, the general entropy production in the system remains positive. Moreover, these processes do not develop gradually, but in leaps and jumps. Due to the emergence of processes with negative entropy production, the total entropy production in a system that has dissipative structures is lower than that of an similar system without them [45]. All other things being equal, the appearance of dissipative structures leads to a decrease in entropy production and wear rate. In the articles that address the principles of maximum and minimum production of entropy, there is no mention of the relationship between these principles and self-organization. Self-organization and the conditions for its occurrence are of particular interest during friction processes, including the machining of metals, such as, for example, cutting. As far as it is currently known, there are no pre-existent signs capable of indicating the possibility of self-organization and the formation of dissipative structures. However, there exists a sufficient condition for the possibility of self-organization—the loss of thermodynamic stability of a system [46,47].

However, to control friction in such a way as to reduce the wear rate, a criterion similar to the principles of minimum or maximum entropy production would be useful. Taking into account the unambiguous relationship between entropy production and wear rate, as well as the decrease in wear rate during self-organization, this article attempts to clarify the behavior of entropy production that ensures the loss of thermodynamic stability in friction systems. Considering that self-organization leads to an abrupt decrease in the wear rate, experimental confirmation of the conclusions obtained in the article will be presented in the form of a dependence of the wear rate on the duration of friction.

## 2. Theoretic Analysis

The process of self-organization in friction system reduces the production of entropy in tribo-films and reduces the wear rate [8]. The fundamental reason for this is that a portion of the frictional energy, which, in the absence of self-organization would have been spent on wear, is instead spent on the formation of dissipative structures in the wake of a self-organization process.

This generally applies for different friction systems such as metal cutting [48,49,50], friction with current collection [51], and plain bearings [52,53]. Self-organization can only occur after a friction system loses thermodynamic stability [46]. The conditions outlined by Lyapunov’s stability theorem are sufficient but not necessary for this to happen [46,47]. 

According to [46,47], the system loses thermodynamic stability when the following holds true:

(1) $$\frac{1}{2}\frac{\partial }{\partial t}\left(\delta^{2}S\right)\;\leq \;0$$

where $\delta^{2}S—$second entropy variation, *t*—time.

According to [46,47]

(2) $$\frac{1}{2}\frac{\partial }{\partial t}\left(\delta^{2}S\right)=\sum_{i}\delta X_{i}\delta J_{i}$$

where *X_i_* and *J_i_* are the corresponding thermodynamic forces and flows.

The right side of (2) shows the excess production of entropy—defined as the variance of thermodynamic flows and forces from the stationary state. According to [46,47], *δ*^2^*S* is the Lyapunov function and it is non-positive:

(3) $$\left(\delta^{2}S\right)\;\leq \;0$$

The system is stable if the below condition is satisfied:

(4) $$\frac{1}{2}\frac{\partial }{\partial t}\left(\delta^{2}S\right)\;\geq \;0$$

As long as condition (4) is satisfied, the system is stable and self-organization is impossible. However, if the derivative of the Lyapunov function is non-positive, i.e., if condition (1) is met, then the system has the opportunity to lose thermodynamic stability, thus, opening the possibility for self-organization. The development of the friction process depends on the pressing force, the relative sliding speed, the compositions, structures and properties of the materials involved, as well as the gaseous or liquid medium (lubricant) and the environment. Considering the entropy of the friction system as a function of time of test (*S*(*t*)), the deviation of entropy from the stationary state can be expressed as follows:

(5) $$\delta S=\frac{\partial S}{\partial t}\delta t+\frac{1}{2}\frac{\partial^{2}S}{\partial t^{2}}{\left(\delta t\right)}^{2}$$

According to [46,47], the second degree term is sufficient in the Taylor series expansion of (5). Entropy variations in (5) occur over the time of the test. In this case, the first entropy variation consists of the sum of entropy production and entropy flow:

(6) $$\frac{\partial S}{\partial t}\delta t=\frac{\partial_{i}S}{\partial t}\delta t+\frac{\partial_{e}S}{\partial t}\delta t=P\delta t+I\delta t$$

where *P* and *I* are the corresponding production and flow of entropy.

The second variation of entropy over time is characterized by the sum of the derivatives of entropy production and flow. If the external conditions (environment, rubbing materials, lubrication, lubrication mode) remain constant, it can be assumed that the entropy flow also remains unchanged over a given time period. Therefore, the time derivative of the entropy flow is zero, and the second entropy variation is characterized by the derivative of entropy production with respect to time:

(7) $$\delta^{2}S=\frac{\partial P}{\partial t}{\left(\delta t\right)}^{2}$$

In this case, the left side of Equations (1) and (4) correspond to the second time derivative of entropy production. The conditions (1) and (3) required for the potential loss of thermodynamic stability and the initiation of a self-organization process are that the first and second derivatives of entropy production must be simultaneously negative.

Let us consider these results for the condition of friction. Once the process of friction has started, entropy production (and as a consequence, wear rate) sharply rises from a state of rest until a steady state is reached, i.e., a localized peak. At a maximum peak of entropy production, the first derivative is zero and the second derivative is negative. If entropy production (as well as wear rate) begins to decrease along a convex curve, then both derivatives become negative, satisfying conditions (1) and (3). If this is the case, then the system may lose thermodynamic stability and self-organization may occur. If entropy production (wear rate) begins to decrease along a concave curve, then the second derivative remains positive and self-organization cannot occur. 

Thus, for a self-organization process to initiate in the first place, it is necessary for entropy production to reach a maximum localized peak and then begin to decrease along a convex curve. Self-organization is a probabilistic process: if conditions (1) and (3) are met, the system has the possibility of losing thermodynamic stability and undergoing self-organization [28,46]. If self-organization does not occur, then the system remains in a state of maximum entropy production and wear rate under the given conditions, until it reaches its natural wear limit. This concurs with [28,54], where it is stated that a high level of energy dissipation is a necessary requirement for the self-organization process. 

Measuring the production of entropy during friction is a rather difficult task. That is why in experimental works, the parameters that characterize entropy production are usually used [46]. Considering that any change in wear rate is directly dependent on entropy production [8], it can be expected that entropy production and wear rate change with time in the same manner. 

The entropy change under friction conditions ($\frac{dS}{dt}$) is as follows [48,51,52]:

(8) $$\frac{dS}{dt}=\frac{dS_{e}}{dt}+\frac{dS_{i}}{dt}\mp \left|\frac{dS_{f}}{dt}\right|-\frac{dS_{s}}{dt}$$

where:

$\frac{dS_{s}}{dt}$ is the entropy change in a friction body due to the wear of its base;

$\frac{dS_{e}}{dt}$ is the entropy flow without the wear;

$\frac{dS_{i}}{dt}$ is the entropy production without friction surface transformations; 

$\frac{dS_{f}}{dt}$ is the portion of entropy production associated with friction surface transformations. 

The term $\frac{dS_{s}}{dt}$ on the right side of expression (8) has a negative sign due to the removal of wear particles from the main body along with their entropy. The negative sign in front of $\left|\frac{dS_{f}}{dt}\right|$ indicates that surface transformations are accompanied by a negative production of entropy. Moreover, it also signifies the passage of self-organization along with the formation of dissipative structures. A plus sign in front of $\left|\frac{dS_{f}}{dt}\right|$ indicates that the surface transformations in tribo-films are accompanied by a positive production of entropy. It also implies that self-organization with dissipative structure formation had not taken place. The sum $\frac{dS_{i}}{dt}\mp \left|\frac{dS_{f}}{dt}\right|$ is the total entropy production, which remains positive according to the second law of thermodynamics.

Under the stationary conditions, the Equation (8) is as follows:

(9) $$\frac{dS_{s}}{dt}=\frac{dS_{e}}{dt}+\frac{dS_{i}}{dt}\mp \left|\frac{dS_{f}}{dt}\right|$$

Under constant external conditions, the value of $\frac{dS_{e}}{dt}$ undergoes negligible changes. Taking into account the additivity of entropy, the value of $\frac{dS_{s}}{dt}$ is proportional to the wear rate. In this case, according to (9) and [9], the wear rate has a direct relationship with entropy production. This makes it possible to evaluate the change in entropy production through the assessment of a change in the wear rate. It follows from (9) and [9], that the wear rate decreases in the aftermath of self-organization. Reduction in the production of entropy and the wear rate could only be accomplished if the friction system had reached the maximum of entropy production prior to the commencement of a self-organization process. The principle of maximum entropy production has, thus, been realized in the friction system.

## 3. Experimental

Below are the dependencies of the wear rate on the duration of friction for three significantly different friction systems (sliding electrical contacts, milling, plain bearings). These systems are united by the fact that an abrupt decrease in the wear rate (self-organization) occurs after reaching the maximum wear rate. This, according to (9) and [9], corresponds to the maximum entropy production.

This article considers the evolution of the following frictional systems: 

A current collection system (the materials used for the friction pair were carbon current collector and copper) [45,48] with a sliding speed of 10 m/s, a clamping force of 40 N, and an electric current of 75 A. Two samples of current-removing materials were pressed against a copper contact wire ring. The diameter of the wire was 11 mm and the diameter of the ring was 0.35 m. The ring was rotated on an axis at a rotational speed of 500 rpm. The contact length of the wire with the current collector material was 10 mm. The hardness of the copper wire was 120 HB. The hardness of the current collection material was 60 HS.

- First, the amount of electric current was increased to determine the value of the current at which a sharp decrease occurred in the intensity of wear (self-organization) and bifurcation. The tests were then carried out at this value of the current (75 A) [48,51]. The weight wear of the current-removing materials was measured every hour;
- A metal-cutting system (end milling of stainless steel using carbide cutting tool with an (Al-Ti)N coating [49]). Semi-finish turning cutting tests were performed. All turning tests were conducted at a cutting speed of 320 m/min, feed rate of 0.2 m/rev, and depth of cut of 1 mm under wet conditions [55];
- A plain-bearing crankshaft system (a friction pair composed of aluminum antifriction alloy steel with lubrication) [47,52]. The tribological tests were carried out based on a shoe-and-roller scheme. The shoe was made of an antifriction aluminum alloy (Al-5.8%Sn-2.7%Pb-4.1%Cu-2.3%Zn-1.5%Mg-1.5%Si-0.03%Ti) while the roller consisted of chromium–nickel steel SNC28 steel (Hakuro Group, Kasai, Japan) was an analogue. The radius of the shoe and the roller was 20 mm, while both of their thicknesses were 10 mm. The steel roller rotated at a speed of 500 rpm. The shoe was pressed against the roller with a force of 167 N. Tests were carried out in API CB oil. The weight wear of the roller and shoe was measured at every hour [52,53].

Attention should be paid to the significant differences between each system. The methods, materials, modes, and experimental conditions are described in the relevant articles [48,49,51,52,53,55].

## 4. Results

**Figure 1:**
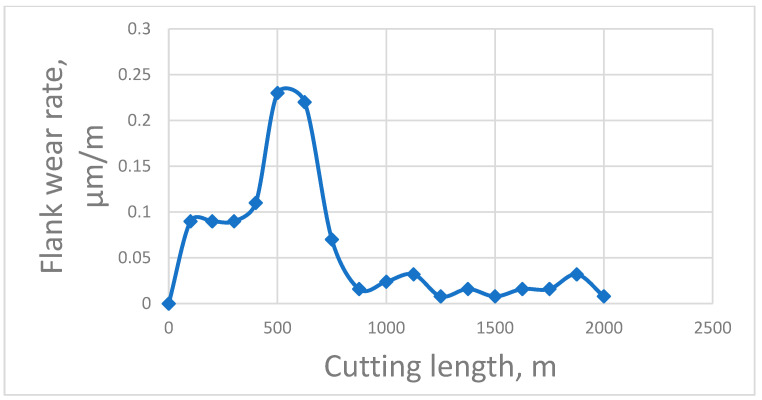
Flank wear rate vs. cutting length.

**Figure 2:**
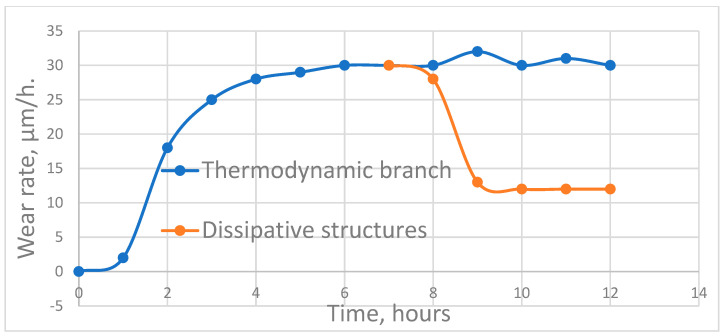
Wear intensity of the carbon current collecting material over time.

**Figure 3:**
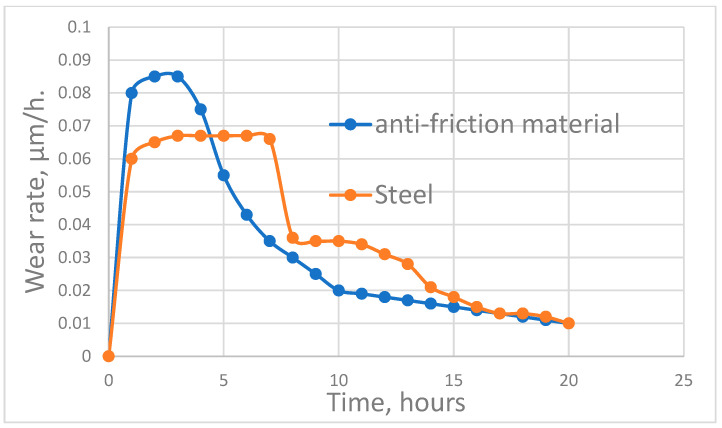
Wear intensity of the antifriction material and steel over time.

It is difficult to establish a direct relationship between entropy production and test times. Figure 1, Figure 2 and Figure 3, instead, show the relationship between wear rate and test time or length of friction. The trend corresponding between wear rate and entropy production is shown in [8,9]. The relatively sharp decrease in wear intensity in Figure 1, Figure 2 and Figure 3 can be attributed in [48,49,51,52,53,55] to the passage of self-organization.

It should be noted that the relationships between wear rate and either time of test or friction length must pass through a maximum peak before self-organization can occur (as evidenced by a sharp decrease in the wear rate). The corresponding relationships of entropy production also follow the same trend.

Figure 2 depicts a bifurcation. The branching curve of wear rate with dissipative structures sharply decreases from the maximum value. If self-organization does not commence at this point, then the magnitude of wear rate (entropy production) remains at the maximum level. It was found that the process of self-organization in tribo-films was accompanied by an abrupt chemical reduction of carbon dioxide by copper. This reaction corresponds to a negative chemical affinity as well as negative entropy production [48,51,56]. Traces of its passage prior to a sharp decrease in the wear rate were not detected. This reaction is a major component in the formation of dissipative structures. 

In Figure 1, the flank wear rate peaks during the first 100 m of cut, but self-organization has not yet occurred. In this system, the wear rate continues to increase until it reaches an even higher maximum, at which self-organization then commences. It is highly likely that the entropy production follows the same trend [8,9]. In this case, self-organization in tribo-films is accompanied by the formation of a high amount of aluminum oxide on the friction surface with predominant mass transfer of either light or heavy elements into the friction zone of the cutting tool, depending on the mode of friction. Prior to friction, there was a uniform elemental distribution on the surface of the tool coating.

Figure 3 shows that when the aluminum alloy of an anti-frictional bearing rubs against steel, a decrease in the wear rate (after reaching a maximum) due to self-organization can be characteristic of both bodies in contact. A system’s wear rate and entropy production during friction can be affected by the intensification of processes occurring on the friction surfaces, such as, for example, seizure. Here, self-organization in tribo-films corresponds with a release of magnesium and zinc from a solid solution based on aluminum and their mass transfer into the friction zone. The release of magnesium and zinc from the solid solution contradicts the corresponding equilibrium state diagrams. Consequently, these processes are accompanied by negative entropy production and are components of dissipative structures. 

Presented below is an example of a self-organization mechanism during the friction of antifriction aluminum alloys. It was previously established that the dissipative structures formed during self-organization are characterized by processes with negative entropy production. These processes are accompanied by an increase in the free energy and, therefore, cannot proceed spontaneously. Such non-spontaneous processes are the result of the interaction of two or more spontaneous dissipative processes [28,46]. Since dissipative structures are characterized by processes with negative entropy production, therefore, all other things being equal, the total production of entropy in systems with dissipative structures is lower than that of equivalent systems without them [45]. The same trend also applies to the rate of wear, according to [10]. 

To determine the mechanisms of dissipative structures, a study was conducted on the tribo-films formed during friction. This study focused on the signature traces left behind by processes with negative entropy production. 

**Figure 4:**
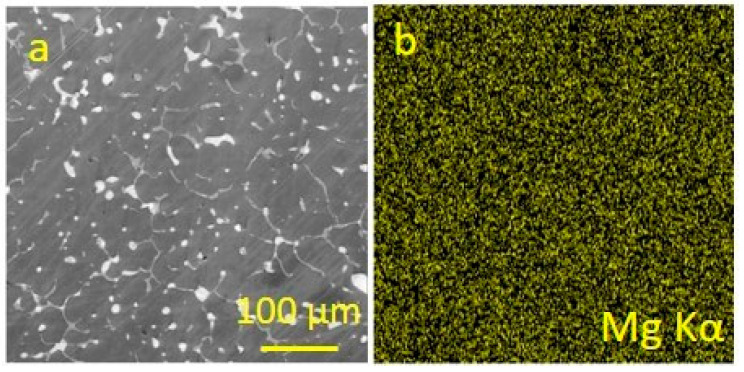
Surface of aluminum antifriction alloy prior to friction: (**a**)—image in secondary electrons; (**b**)—magnesium distribution map.

During the friction of an antifriction complex aluminium alloy [48,52,53] against steel (Figure 3), the negative entropy production process was the mass transfer of magnesium into the friction zone and its release from an aluminum-based solid solution. Figure 4 shows the EDX of SEM images of the alloy surface and the characteristic distribution of magnesium in the alloy prior to friction. It follows from Figure 4b that magnesium is evenly distributed within the alloy and becomes dissolved in its aluminum base.

**Figure 5:**
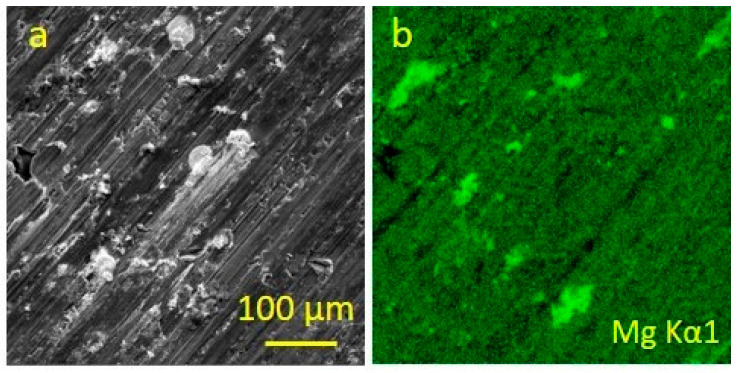
The friction surface of aluminum antifriction alloy: (**a**)—image in secondary electrons; (**b**)—magnesium distribution map.

Figure 5 shows the surface of the alloy after friction [57]. In Figure 5b, it is evident that the distribution of magnesium after friction becomes uneven and there is a segregation of magnesium from the aluminum-based solid solution. 

**Figure 6:**
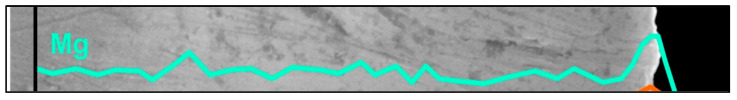
Concentration profile of magnesium in a plane perpendicular to the friction surface at a length of 50 μm. The friction surface is on the right.

Figure 6 shows the concentration profile of magnesium in a plane perpendicular to the friction surface. It follows from Figure 6 that magnesium diffuses to the friction surface during friction, at which point it is released from the solid solution. During the process of friction, magnesium diffuses in the direction of increased concentration.

The temperature on the friction surface did not exceed 130 °C (oil flash point). According to the Al–Mg state diagram, the solubility of magnesium in aluminum increases with temperature and is about 5% wt. at a temperature of 130 °C [58]. The alloy contains 1.5% wt. Mg. Therefore, the release of magnesium from a solid aluminum-based solution contradicts the equilibrium state diagram. As a consequence, this process is directed towards an increase in the free energy and is accompanied by a negative entropy production. Diffusion of magnesium that results in its uneven distribution and the release of magnesium from a solid aluminum solution are both examples of dissipative structures.

If self-organization does not occur at a point of maximum entropy production (energy dissipation) and wear rate, then the entropy production of a friction system continues to grow until it reaches an even greater potential maximum, at which point the probability of self-organization also increases (Figure 1). If the friction system does not have such an opportunity, then the entropy production and wear rate remain at the maximum level (Figure 2, thermodynamic branch). Given the significant differences between friction systems, it can be concluded that maximum entropy production is a condition for the initiation in tribo-films of a self-organization process capable of substantially decreasing the wear rate. This conclusion is important from the point of view of friction mode selection. For example, it implies the possibility of applying rigid running-in modes for frictional bodies, possibly in a pre-seizure state. This can lead to a decrease in the running-in period as well as the time it takes for self-organization to initiate, which leads to a decrease in the overall wear. 

## 5. Conclusions

Before a possible loss of thermodynamic stability and possible self-organization of tribo-films, entropy production must first reach a maximum peak and then begin to decrease along a convex curve. If self-organization does not occur when the maximum entropy production is reached, then the friction system strives to reach an even higher maximum (provided that the system has such a possibility), increasing the probability of self-organization. If the friction system does not have the possibility of transitioning to an increased level of maximum entropy production, then both the entropy production and wear rate remain at their current maximum, which results in systemic failure. This demonstrates the relationship between the principle of maximum entropy production and the occurrence of self-organization of tribo-films in friction systems.

A possible description of the behavior of entropy production using the wear rate facilitates the application of the principles of non-equilibrium thermodynamics and the theory of self-organization for friction units and materials. 

## Data Availability

All data generated or analyzed during this study are included in this published article. Additional raw data collected are, however, available from the authors upon reasonable request.
